# Radiological Insights into Acellular Dermal Matrix Integration in Post-Mastectomy Breast Reconstruction: Implications for Cancer Patient Management

**DOI:** 10.3390/cancers17060933

**Published:** 2025-03-10

**Authors:** Luciano Mariano, Andrea Lisa, Luca Nicosia, Anna Carla Bozzini, Sergio Miranda, Manuela Bottoni, Valeria Zingarello, Filippo Pesapane, Mario Rietjens, Enrico Cassano

**Affiliations:** 1 Breast Imaging Division, IEO European Institute of Oncology, IRCC, 20141 Milan, Italy; anna.bozzini@ieo.it (A.C.B.); filippo.pesapane@ieo.it (F.P.); enrico.cassano@ieo.it (E.C.); 2 CDI Italian Diagnostic Center, Via Saint Bon 20, 20147 Milan, Italy; 3 Department of Plastic and Reconstructive Surgery, IEO European Institute of Oncology, IRCCS, 20141 Milan, Italy; andreavittorioemanuele.lisa@ieo.it (A.L.); sergio.miranda@ieo.it (S.M.); manuela.bottoni@ieo.it (M.B.); valeria.zingarello@ieo.it (V.Z.); mario.rietjens@ieo.it (M.R.); 4 Department of Medical Biotechnology and Translational Medicine (BIOMETRA), Reconstructive and Aesthetic Plastic Surgery School, University of Milan, Via Festa del Perdono 7, 20122 Milan, Italy; 5 Department of Surgical Sciences, University of Rome “Tor Vergata”, Cracovia n. 50, 00133 Rome, Italy

**Keywords:** breast cancer, acellular dermal matrix, breast reconstruction, breast imaging

## Abstract

Background: Breast reconstruction (BR) following mastectomy plays a critical role in restoring breast contour and improving patients’ quality of life. Acellular dermal matrices (ADMs) have emerged as valuable adjuncts in BR, providing structural support and enhancing soft tissue integration. However, their radiological characteristics remain underexplored, leading to potential misinterpretation and diagnostic challenges. This study aims to evaluate the imaging features of ADM in post-mastectomy patients using conventional imaging modalities, identifying its temporal evolution and clinical implications for radiologists and surgeons. Materials and Methods: This single-centre retrospective study included breast cancer patients who underwent mastectomy followed by ADM-assisted BR. Patients were monitored using standardised radiological follow-up protocols, including digital mammography (DM) and ultrasound (US), at 6 (T0), 12 (T1), and 18 months (T2) postoperatively. The primary outcomes assessed were the presence and evolution of ADM-related imaging findings, differentiation between normal ADM integration and pathological changes, and the role of different imaging modalities in ADM evaluation. Results: Sixty-three patients met the inclusion criteria and underwent radiological follow-up. At T0, ADM was identified in 16% of cases, primarily as a peri-capsular hypoechoic thickening on US and a linear peri-implant density on DM. At T1, these findings were partially resolved, with 11% of cases still displaying peri-capsular changes. By T2, imaging signs of ADM were further reduced, with only 7% of cases showing residual peri-capsular thickening or pseudonodular formations. No ADM-related complications, graft rejection, or implant loss were detected. These findings suggest a progressive integration of ADM into the host tissue over time, with characteristic imaging changes that must be recognised to avoid misdiagnosis or unnecessary interventions. Conclusions: ADM exhibits a dynamic radiological evolution in post-mastectomy BR, with its imaging characteristics gradually fading. Recognising these features is critical for radiologists and surgeons to ensure accurate interpretation and optimised patient management. A structured imaging follow-up protocol, incorporating US as the primary modality and MRI in cases of inconclusive findings, is recommended to improve diagnostic accuracy. Future multicentre studies with extended follow-up and advanced imaging techniques are necessary to refine radiological criteria and further explore ADM integration patterns. A multidisciplinary approach is essential to enhance clinical decision-making, reduce unnecessary interventions, and optimise patient outcomes in ADM-assisted BR.

## Simple Summary

Breast reconstruction (BR) following mastectomy is a critical step in breast cancer treatment, improving both aesthetic outcomes and patient well-being. Acellular dermal matrices (ADMs) are widely used in BR to provide structural support and promote tissue integration. However, their radiological characteristics remain underexplored, leading to potential misinterpretations. This study evaluates the imaging features of ADM over time using conventional imaging techniques such as digital mammography (DM) and ultrasound (US). Our findings show that ADM undergoes a progressive integration process, with specific imaging changes gradually fading. Recognising these patterns is essential for radiologists and surgeons to differentiate normal ADM evolution from pathological findings, avoiding unnecessary biopsies or interventions. A structured imaging follow-up protocol, with US as the primary modality and MRI reserved for inconclusive cases, can enhance diagnostic accuracy. A multidisciplinary approach is key to optimising patient management and ensuring the best clinical outcomes in ADM-assisted BR.

## 1. Introduction

Breast cancer (BC) is the most commonly diagnosed cancer worldwide, including low- and middle-income countries [1]. A recent reduction in mortality of about 6% and a current 5-year survival rate of 87% are attributed to efficient screening programs and advances in diagnosis and therapy [2]. Several factors, including tumour location, size, mammographic features, and radiotherapy contraindications, influence the choice between a surgical conservative approach and mastectomy, with the latter generally used for extensive disease, inflammatory BC, or patient preference [3,4].

Breast reconstruction (BR) post-mastectomy is an essential step for BC patient treatment, offering high oncological safety and better aesthetic outcomes [5,6,7]. BR can involve alloplastic materials, autologous tissues, or both and may be carried out either immediately (BRi) or at a late stage (BRd) [8,9,10]. BRi is widely recommended and considered a quality indicator by EUSOMA, with a minimum standard of 40% [11]. BRi techniques may include two-stage (tissue expansion followed by the implant, FTI) or one-stage (direct-to-implant, DTI), with a recent interest in subcutaneous implant placement [8]. Despite slightly higher skin flap necrosis risks with DTI [12,13], comparable outcomes with prepectoral placements have been observed [14,15].

Acellular dermal matrix (ADM) is an innovative approach in BR involving biotechnologically engineered tissue from human, bovine, or porcine sources, processed to remove the epidermis by a sequential decellularisation process [16,17,18,19], preserving its collagen and elastin scaffold to promote muscle stabilisation and provide soft tissue support [20,21]. ADM’s advantages include maintaining normal chest wall anatomy, reducing postoperative pain and lowering capsular contracture rates [22,23]. However, although its use may increase the risk of infection, seromas, and skin flap necrosis, overall complication rates are often like non-ADM procedures [24,25,26,27,28]. A review from the Mastectomy Reconstruction Outcomes Consortium (MROC) found no significant increase in complication rates, time to exchange, or patient-reported outcomes with ADM use [26].

Despite its advantages, the lack of Food and Drug Administration (FDA) approval for ADM use in BR raises regulatory and clinical adoption challenges, which must be critically evaluated to ensure patient safety and standardised protocols.

To date, the available literature describing the radiological features of ADMs remains underexplored and limited to a few studies. However, recognising their imaging presentation is paramount in clinical practice, as misinterpretation may lead to unnecessary follow-up investigations, unwarranted biopsies, or even misdiagnosis of disease recurrence. Given that ADM integrates into the host tissue over time, its imaging characteristics can evolve, making it crucial for radiologists and clinicians to differentiate regular post-surgical changes from pathological findings. Additionally, recognising the expected evolution of ADM across different imaging modalities can optimise follow-up strategies, reducing patient anxiety and healthcare costs.

This preliminary study aims to bridge the existing knowledge gap by evaluating the radiological characteristics of ADM across conventional imaging techniques at multiple postoperative intervals. By providing a detailed analysis of its evolution over time, we seek to establish reliable imaging criteria that can facilitate differentiation from residual disease, recurrence, or post-BR complications. Furthermore, we discuss the implications of imaging modality selection, including radiation exposure and clinical utility considerations, to refine future diagnostic protocols and enhance decision-making in post-mastectomy reconstruction follow-up.

## 2. Materials and Methods

### 2.1. Study Design and Population

According to the Declaration of Helsinki guidelines and with approval from the Ethics Committees of the European Institute of Oncology in Milan (protocol number UID 4231), we conducted a single-centre retrospective study starting in November 2022. This study included BC patients who underwent mastectomy followed by BR using ADM.

Informed consent was obtained from all participants before their inclusion in the study.

All enrolled patients underwent a standardised radiological follow-up using conventional imaging techniques (digital mammography, DM, and/or ultrasound, US) at our Breast Imaging Division for up to 18 months post-surgery. This time frame was selected as it is considered appropriate for the full ADM integration into the patient’s biological tissues. To ensure consistency in follow-up assessments, imaging was performed at 6 months (T0), 12 months (T1), and 18 months (T2), following internal clinical recommendations for post-mastectomy monitoring. Patients who did not complete radiological follow-up at our institute or underwent BR without ADM were excluded from the study.

### 2.2. Acellular Dermal Matrix (ADM) and Surgical Technique

In our clinical study, we adopted Native^®^ ADM, a 0.6 mm thick, rectangular-shaped continuous-surface collagen matrix derived from pig dermis used as a tissue source. The material undergoes a deantigenation treatment to remove all animal cells and obtain a pure extracellular matrix, ensuring biocompatibility and eliminating rejection risk. The ADM is not cross-linked and supplied without preservatives; it has been lyophilised for optimal preservation and is sterilised with ethylene oxide.

The surgical technique employed in this study for ADM implantation was the dual-plane subpectoral approach [29]. This method involves the creation of the subpectoral pocket, followed by inferior-medial elevation and precise division of the pectoralis major muscle from its inferior margin. The ADM is sutured to the inferior portion of the pectoralis major and extends to the lateral mammary fold without elevating the serratus anterior muscle. The implant is placed below the pectoralis-ADM layer, with the ADM attached to the inframammary fold, ensuring complete coverage and a hand-in-glove fit. Subcutaneous and subpectoral suction drains are then placed before skin closure.

### 2.3. Image Acquisition

Patients underwent a comprehensive conventional imaging follow-up to ensure thorough post-surgical monitoring and evaluation. The follow-up protocol included the following:

- DM with integrated tomosynthesis acquisition: performed on the controlateral breast at least one year after the last DM and at least six months following any radiation therapy. In cases of patients who underwent nipple-sparing mastectomy with clinical and/or US suspicion arose, DM was also conducted on the reconstructed breast to assess the retroareolar and periprosthetic regions;
- Bilateral breast US: conducted every six months after surgery, with additional evaluation of the axillary, subclavicular, and first internal mammary chain lymphatic stations.

DM was acquired in cranio-caudal (CC) and medio-lateral-oblique (MLO) views, using the GE^®^ Healthcare, Senographe Pristina^®^ system (Chalfont St. Giles, UK). Tomosynthesis was performed exclusively in the MLO view. Radiation dose values remained below 2.5 mGy per view, as recommended by the European guidelines for screening mammography [30] and the Mammography Quality Standards Act guidelines [31].

US examination was conducted bilaterally using a Canon Diagnostic Ultrasound System—Aplio i800 scanner (Tokyo, Japan), equipped with 5–18 MHz linear probes specifically optimised with presets dedicated for breast parenchyma evaluation. Particular attention was given to the implant assessment to detect signs of periprosthetic fluid collection or rupture. Additionally, lymphnode evaluation was systematically performed in the axillary, subclavicular, and early internal mammary chain regions to monitor potential post-surgical or oncological developments.

### 2.4. Image Interpretation and Data Analysis

DM and US examinations were performed and evaluated by dedicated radiologists with over five years of experience in breast imaging. During the assessment, the radiologists were informed of the patient’s surgical history, medical background, and any post-surgical complications to ensure accurate interpretation.

Patients were stratified into groups based on the type of surgery received. US and/or DM images were analysed at 6 months (T0), 12 months (T1), and 18 months (T2) post-surgery to ensure consistent evaluation of ADM integration, focusing on the presence of ADM-related radiological signs, their temporal evolution, and the occurrence of recurrence or complications.

Imaging features obtained through conventional techniques were evaluated based on breast density, lesion site, size, lesion type, margins, and BI-RADS classification [32].

Data collection and analysis were conducted using a descriptive statistical approach, with results presented as percentages to provide a comprehensive and accurate overview of the findings. Inferential statistical methods were not applied due to the limited sample size, which restricted statistical power and increased the risk of non-representative or non-significant outcomes in inferential analyses.

## 3. Results

The initial study included 91 patients who underwent a mastectomy and BR with ADM; 28 patients were excluded based on the study criteria.

Radiological follow-up images were reviewed for the remaining 63 patients. Among them, twenty patients underwent delayed BR after unilateral mastectomy, with expander placement and subsequent implant reconstruction with ADM (eight nipple-sparing and twelve total mastectomies), while forty-three patients underwent immediate implant-based reconstruction after mastectomy with ADM placement. Of these, nine had mastectomy with immediate implant reconstruction and ADM (five nipple-sparing and four total mastectomies), and thirty-four patients initially underwent mastectomy with immediate implant reconstruction without ADM but later required implant replacement with ADM due to complications (nineteen nipple-sparing and fifteen total mastectomies)

**Figure 1:**
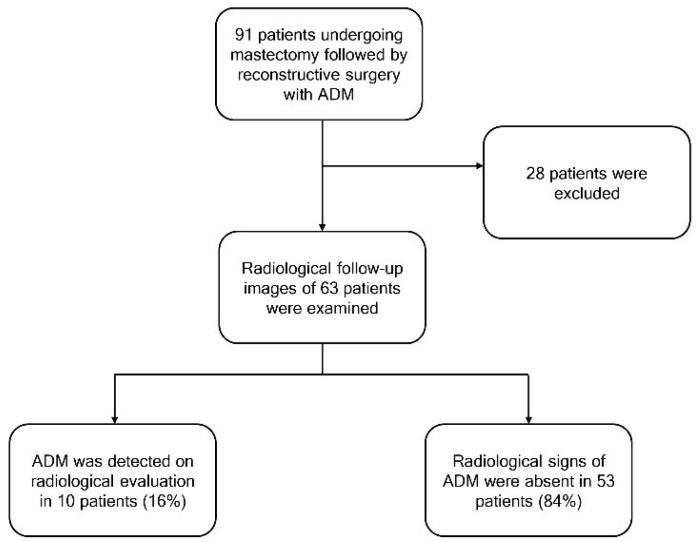
Flowchart of the study.

The study flowchart and the characteristics of patients who underwent ADM placement are presented in Figure 1 and Table 1, respectively.

All 63 patients underwent radiological follow-up using conventional imaging techniques (DM and/or US) for up to 18 months post-surgery. Five patients received US-only follow-up due to bilateral total mastectomy.

At the first post-surgical evaluation, radiological signs of ADM were absent in 53 patients (84%), as confirmed by the US. ADM was detected in ten patients (16%): eight through US only and two through both DM and US. Among them, two had undergone immediate implant BR after mastectomy, three had undergone delayed BR after mastectomy, and five had initially undergone mastectomy with immediate implant reconstruction (without ADM) but later required implant replacement with ADM due to complications.

**Figure 2:**
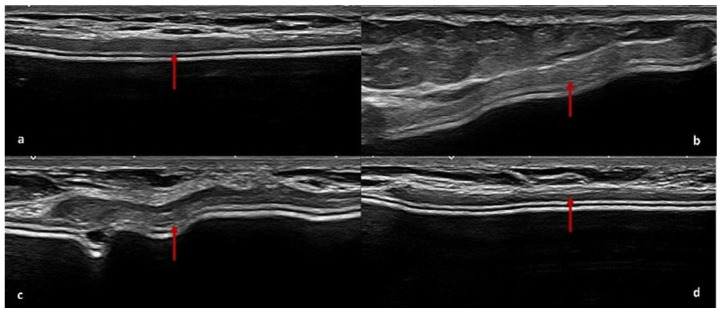
ADM features on US. ADM is observed as a peri-capsular hypoechoic thickening (red arrows) on the implant profile (**a**–**d**).

**Figure 3:**
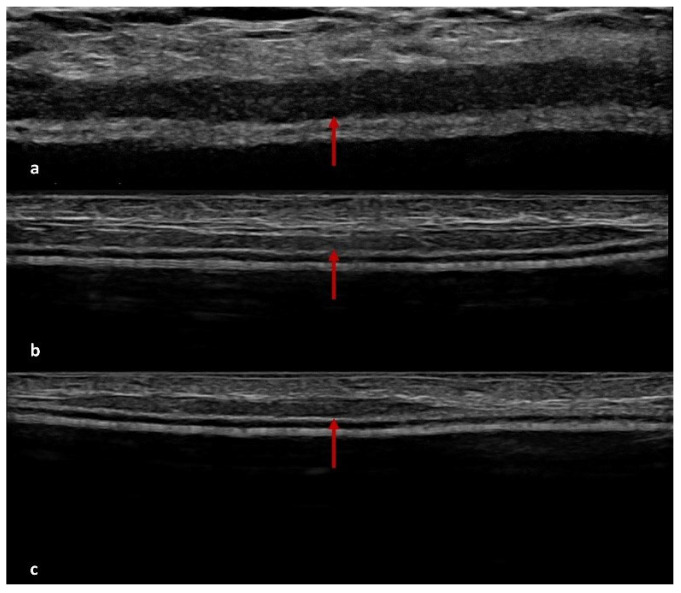
ADM features on US. The US scans of patients undergoing nipple-sparing mastectomy show a homogeneous hypoechoic peri-implant thickening (red arrows) at 6 months (**a**), gradually reduced at 12 (**b**) and 18 (**c**) months by ADM integration in the soft tissues of the host.

**Figure 4:**
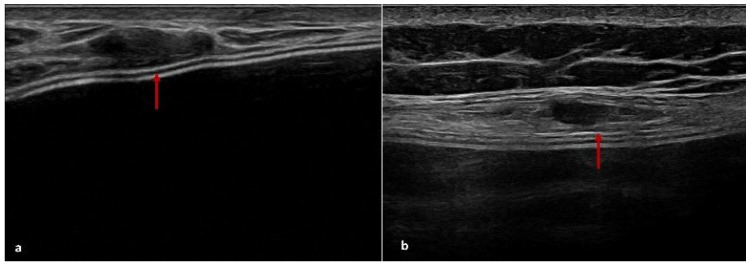
ADM features on US. A focal hypoechogenic lesion on the implant profile (red arrows) may be a normal finding post-BR with implant and ADM (**a**,**b**).

At T0 (6 months post-surgery), the US detected hypoechoic peri-capsular thickening (up to about 2 mm) in ten patients; at T1 (12 months post-surgery), the US showed persistent peri-capsular thickening in seven patients, while three patients exhibited a reduction in capsular thickening compared to the first evaluation; at T2 (18 months post-surgery), five patients still exhibited peri-capsular thinking on US, three patients no longer showed recognisable ADM, and two patients developed more evident peri-capsular pseudonodular images, likely related to ADM (Figure 2, Figure 3 and Figure 4).

**Figure 5:**
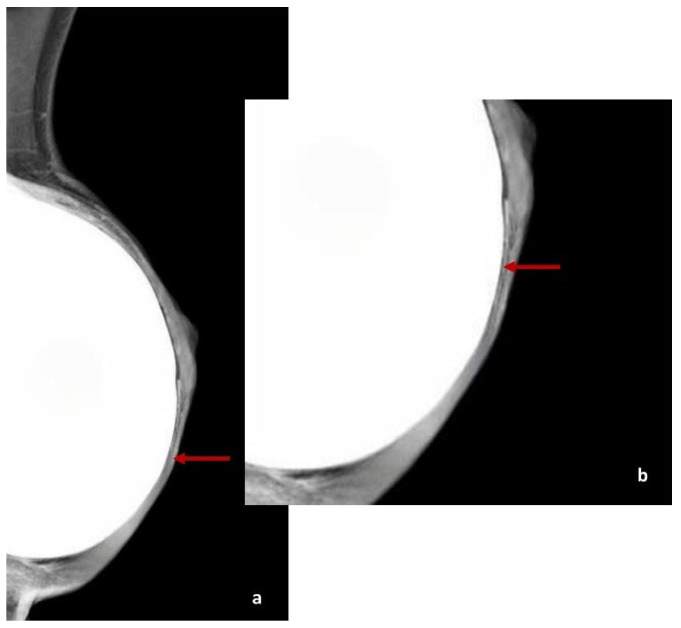
ADM features on DM. The medio-lateral-oblique DM view (**a**) and the targeted magnification (**b**) of the left breast approximately six months after nipple-sparing mastectomy with implant reconstruction and placement of porcine-derived ADM show a linear thickening (red arrow) on the lower profile.

**Figure 6:**
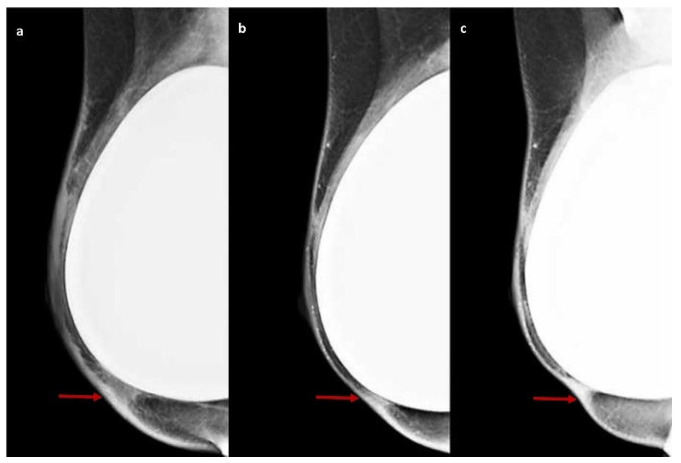
ADM features on DM. The medio-lateral-oblique DM views of the right breast after nipple-sparing mastectomy with implant reconstruction and placement of porcine-derived ADM show a peri-implant linear thickening (red arrows) at six months (**a**), reduced at 12 months (**b**) and completely disappeared at 18 months (**c**).

Only three patients underwent DM of the operated breast: one patient showed no radiological signs of ADM; two patients exhibited peri-implant linear thickening at T0, which completely disappeared at T1 in one case and reduced at T1 and almost completely disappeared at T2 in the other case (Figure 5 and Figure 6).

A summary of cases presenting radiological signs of ADM is provided in Table 2.

## 4. Discussion

Integrating ADM in BR has revolutionised surgical practice, providing significant advantages in aesthetic outcomes and patient recovery after mastectomy. Since it was introduced by Dieterich [33] and Salzberg [34] in 2001, ADM has been widely accepted traction in BR due to its ability to reduce complications associated with traditional techniques, particularly capsular contracture in patients undergoing post-mastectomy radiotherapy [22,23,24,25,26,27,33,34,35]. Furthermore, ADM has facilitated immediate one-stage BRs, notably in patients over 65, promoting a faster and less arduous postoperative recovery [36]. ADM has been successfully employed in our centre to increase the thickness of irradiated subcutaneous tissues, significantly improving clinical outcomes [37].

ADM is an allograft (derived from human donors) or xenograft (from animal sources) designed to act as a biological scaffold for fibroblasts and angioblasts’ growth, gradually replaced by host connective tissue at varying rates depending on the clinical context [20,21]. Initially utilised in the reconstruction of cutaneous defects, including burn wounds and abdominal wall defects, ADM has become widely adopted in BR, where it plays an essential role in providing adequate soft tissue support to the mastectomy skin flaps. Its application varies depending on the surgical approaches: ADM stabilises the pectoral muscle in the retropectoral BR technique, preventing the upward migration phenomenon commonly called “window shading”. Conversely, in the prepectoral approach, ADM lines the inner surface of the mastectomy skin flap, providing additional structural support and enhancing tissue integration [38].

Despite its widespread use in BR, the FDA approved ADM use in this setting as off-label [39]. However, its adoption in more than 60% of alloplastic BR procedures in the United States since 2005 underscores the clinical community’s confidence in its safety and efficacy [40]. The use of ADM should always be preceded by a thorough discussion between the surgeon and the patient, carefully weighing the risks and benefits before surgery [39]. In this regard, in March 2021, the FDA issued recommendations for patients and healthcare providers, emphasising that before surgery, patients should be informed about the benefits and risks of implant-based BR with and without ADM and that if ADM is used, the specific type should be discussed with the healthcare provider. In addition, it is essential to recognise that although ADM is used for various kinds of reconstruction, its application in BR remains off-label, and prior surgical removal of implanted ADM is not recommended [41]. Finally, in the event of complications following BR with ADM, it is encouraged to report them through MedWatch, the FDA’s safety information and adverse event reporting program [42].

The increased use of ADM has sparked interest in delineating its radiological features in patients undergoing post-mastectomy reconstruction, presenting a new frontier for breast radiologists. Familiarity with the main features of ADM in radiological assessment is essential for accurately discerning its tissue integration, surgical complications, and residual or recurrence disease.

Several studies have explored the possible radiological aspects of ADM, shedding light on its imaging features in various clinical settings. Kim reported a case study where ADM presented as an elongated thickening along the implant profile on DM and as a soft tissue density mass on subsequent non-contrast tomography in a patient with a palpable periprosthetic nodule [43]. Similarly, Buck et al. also described a remarkable case involving a woman who developed a new palpable mass following mastectomy. Surgical excision revealed an inflammatory giant cell infiltrate attributed to the presence of ADM [44]. Furthermore, in a study examining tissue biopsies obtained one year after BR with ADM, the pathological analysis demonstrated almost complete integration of ADM into the patient’s native tissue [45]. Overall, these findings highlight the critical importance of accurately interpreting suspicious lesions or peri-implant formations in patients with a history of ADM use, allowing differentiation between regular ADM integration, residual disease, and recurrence.

Radiologists need to be aware that ADM reabsorption and biointegration is a dynamic process among inflammation (0–3 days), increased oxygen consumption and neoangiogenesis at the ADM border (10–14 days), and vascular and inflammatory cell penetration into the centre of ADM (>21 days), and thus ADM appearance change during follow-up, requiring careful interpretation [46].

For surveillance of women undergoing BR, there are no standardised guidelines for follow-up imaging post-mastectomy. A meta-analysis including 16 studies reported low detection rates of occult malignancies in the three primary imaging modalities (DM, US, and MRI), failing to support a definitive role for routine imaging after mastectomy [47]. Because the only place where cancer can occur is either right below the skin in the subcutaneous tissue or just over the pectoralis muscle, according to the 2015 American Society of Clinical Oncology (ASCO) guidelines, clinical examination remains the cornerstone of oncologic surveillance in these patients, and other imaging modalities should be used only as adjuncts to clarify any physical findings [48]. However, this approach is arbitrary, as no study has evaluated the effectiveness of individualised follow-up programs based on a patient’s specific risk profile [49].

In 2019, the FDA recommended starting breast implant imaging with magnetic resonance imaging (MRI) or US five to six years after implantation, with follow-up every two to three years [50]. However, there is little evidence to support routine or yearly MRI imaging because it is expensive and may lead to anxiety and false positive results, resulting in unnecessary surgery [51,52]. A meta-analysis of women undergoing mastectomy and BR reported a cancer detection rate of 4.7 cases per 1000 MRI screening exams, but only 1.1 per 1000 were clinically occult [47]. Similarly, US for surveillance is not recommended. In a systematic review, routine US screening in mastectomy patients detected cancer in 2.7 cases per 1000 exams (0.3%), with most cases palpable, suggesting limited value in asymptomatic patients [47].

Routine DM is also technically limited in patients with breast implants and is generally not recommended. Despite the lack of consensus, some institutions perform DM of breasts undergoing nipple-sparing mastectomy, as residual tissue in the areola-nipple complex may rarely give rise to a new neoplasm. However, the clinical value of this approach remains uncertain and probably limited.

In our institutional protocol, we prefer that patients who undergo mastectomy with BR undergo biannual US evaluation to assess any post-surgical complications and implant integrity. In addition, we perform an annual DM of the contralateral unmastectomised breast, and if US is suspected, also of the breast undergoing nipple-sparing mastectomy. MRI is added to the diagnostic workup for patients with suspicious and inconclusive findings on conventional imaging. After the first two years, follow-up continues with routine surveillance adapted to the patient’s risk profile.

The results of our study provide valuable insights into the radiological evolution of ADM integration over time, emphasising the dynamic changes observable through DM and US. Reviewing conventional radiological investigations performed at our Breast Imaging Division on patients undergoing mastectomy with BR and ADM use, we observed that the main features of ADM on US and DM were a homogeneous hypoechoic peri-capsular thickening, up to approximately 2 mm, on US, and a linear radiopaque peri-implant thickening at DM. In support of our results, the following radiological features identifying the presence of ADM are reported from the literature data.

### 4.1. DM Features of ADM

According to literature data, ADM may exhibit distinct features on DM following BR. These characteristics may be identified in standard imaging views, such as CC and MLO, and when using specialised techniques such as the implant retraction manoeuvre (Eklund view). ADM often appears as a layer of increased density adjacent to the implant profile or, alternatively, as a well-defined, iso-dense thickening. The size of this thickening may vary compared to normal glandular tissue. These radiological features, however, are not always specific and can occasionally mimic other conditions, such as seromas or post-surgical hematomas [53].

Over time, the mammographic visibility of ADM tends to decrease as the material integrates into the host tissue and surrounding structures. This temporal evolution highlights the dynamic nature of ADM’s radiological presentation in post-surgical follow-up [53,54,55].

### 4.2. US Features of ADM

US provides valuable insights into the evolution of ADM characteristics post-surgery. Initially, ADM appears as a thin hypoechoic layer surrounding the implant, measuring approximately 0.3 to 0.6 mm at one month, which may thicken to 3 mm over time. A transient fluid collection between the ADM, implant, and subcutaneous tissue is often observed in the early postoperative period, gradually resolving as integration progresses. As the ADM assimilates into the surrounding tissue, it contributes to a thickened layer between the implant and the overlying skin [56,57]. In some cases, ADM may present as a focal hypoechoic area contiguous to the implant profile. This layer tends to be thinner over convex regions but may fold at the medial or lateral margins, forming nodular structures that could be misinterpreted as pathological [57].

Kim et al. correlated US findings with histopathology, identifying focal hypoechoic thickening as a sign of chronic inflammation, while diffuse echogenicity was linked to myofibroblast proliferation and collagen deposition. Hyperechoic spots within ADM have been attributed to small air bubbles introduced during implantation, as confirmed histologically [56].

Over time, ADM integrates fully into the host tissue, gradually losing its distinct US features. This process reflects its high biocompatibility and seamless adaptation within the tissue environment [57].

Understanding ADM’s imaging characteristics is important for radiologists and surgeons, as they directly impact clinical decision-making in post-mastectomy BR follow-up. Radiologists must accurately differentiate ADM-related imaging findings from potential signs of disease recurrence, ensuring a precise interpretation that prevents unnecessary interventions or missed diagnoses. A comprehensive radiological assessment is essential for surgeons to guide postoperative management, monitor ADM integration, and anticipate potential complications. Given that ADM undergoes a progressive integration process, its imaging features evolve over time, requiring a structured and standardised follow-up protocol. Based on our findings, we propose that patients undergoing ADM-based BR be monitored through a multimodal imaging approach: US should be the primary modality for early post-surgical assessment, particularly for identifying peri-capsular thickening or fluid collections, while DM remains valuable in patients with nipple-sparing mastectomy. In cases where persistent ADM-related alterations are detected beyond 12 months post-surgery or where imaging findings are inconclusive, MRI should be considered to provide a more detailed evaluation of vascularisation and inflammatory changes. Clinically, stable ADM-related findings require routine surveillance, while persistent nodular formations or increasing peri-capsular thickening should prompt further evaluation, including biopsy, when warranted [46]. The collaboration between radiologists, surgeons, and pathologists is essential to optimise patient outcomes, ensuring that imaging findings accurately correlate with clinical presentation and surgical history. Establishing structured radiological reporting criteria and decision-making frameworks will enhance diagnostic confidence, reduce unnecessary interventions, and improve the overall management of patients undergoing ADM-assisted BR.

Despite the promising insights, several challenges and limitations of our study warrant discussion. First, the relatively small sample size reduces the generalizability of our results. The uneven duration of follow-up among patients compounds this limitation, as many underwent BR only recently, allowing only one postoperative imaging evaluation. These limitations underscore the need for more extensive, multicentric studies with longer follow-up periods to validate and extend our results. However, we aim to characterise ADM’s radiological features and its temporal evolution, offering preliminary data to support future research.

Another crucial area to explore involves comparing radiological features between ADMs derived from different sources, such as human, bovine, or porcine origin. Variations in processing techniques and biological composition could influence ADM behaviour, potentially producing different imaging characteristics. Investigating these differences could enhance radiologists’ ability to interpret ADM-specific findings accurately and tailor their evaluations to the material used. A recent study demonstrated histological and clinical differences between human and porcine-derived ADM in BR. Specifically, human ADM shows greater fibroblast and capillary growth and a lower inflammatory response than porcine ADM [58]. These biological variations may impact the imaging appearance, potentially affecting the degree of contrast enhancement, the presence of inflammatory changes, and the long-term integration. Despite these differences, both human and porcine ADM demonstrated clinical efficacy, with low complication rates and no graft rejection or implant loss cases in long-term follow-up [58]. Understanding these differences is critical for refining radiologic evaluation criteria and optimising patient-specific reconstructive strategies.

Furthermore, while our study focused exclusively on conventional imaging techniques, DM and US, expanding the analysis to include advanced modalities such as MRI would provide a more comprehensive understanding of ADM’s radiological profile. With its superior soft tissue contrast and capability to assess vascularisation and inflammatory changes, MRI could offer valuable complementary information, particularly in complex cases with inconclusive DM and US findings.

In addition to imaging advancements, an emerging area of interest involves leveraging advanced electrokinetic techniques to study cellular interactions with ADM. Recent developments, such as the hydroelectric actuator described by Moharamipour et al., make it possible to analyse cancer cells’ electrophoretic and dielectrophoretic behaviours under controlled conditions. These methods highlight how cellular surface charge and motility can be influenced by electric fields, offering a unique perspective on tissue integration and interaction [59]. Such technologies pave the way for future investigations into ADM’s cellular dynamics and integration with host tissues, potentially optimising its clinical application.

## 5. Conclusions

This study provides a comprehensive analysis of the radiological evolution of ADM integration in BR, highlighting the main imaging features observed over time using conventional modalities such as DM and US. Our results demonstrate that ADM undergoes a progressive biointegration process, with characteristic radiological changes that may aid in distinguishing normal postoperative evolution from pathological findings. Understanding these imaging characteristics is critical for radiologists and surgeons, as it directly impacts clinical decision-making, post-mastectomy surveillance, and patient management.

Radiologists must be able to recognise expected imaging changes of ADM over time to avoid misinterpretations that could lead to unnecessary biopsies or diagnostic delays. Implementing standardised imaging protocols, with clear distinctions between expected findings and potential complications, is essential to improve diagnostic accuracy. From a surgical perspective, accurate radiological assessment of ADM allows optimisation of postoperative follow-up, early detection of complications, and more accurate surgical planning for any necessary revisions. Therefore, a multidisciplinary approach involving radiologists, surgeons, and pathologists is critical to ensuring optimal management of patients undergoing ADM-assisted BR.

Future multicentric studies with extended follow-up and larger cohorts will be essential to validate these findings and refine imaging criteria for ADM evaluation. Additionally, integrating advanced imaging techniques such as MRI could provide further information into ADM vascularisation and inflammatory response, improving diagnostic accuracy in complex cases.

Beyond imaging, emerging electrokinetic techniques could offer novel perspectives on ADM–host tissue interactions, with potential applications for optimising its clinical use. Adopting standardised imaging protocols and shared decision-making frameworks between radiologists and surgeons will improve diagnostic safety, reduce unnecessary interventions, and ensure the best possible patient outcomes.

## Figures and Tables

**Table 1:** Characteristics of patients who underwent ADM placement.

Clinical Presentation	Number of Patients Undergoing ADM Placement
Previous mastectomy and expander reconstruction	20
Previous mastectomy and immediate implant reconstruction with ADM	9
Previous mastectomy and immediate implant reconstruction with subsequent ADM	34
Total	63

**Table 2:** Cases with conventional radiological signs of ADM.

Technique	ADM Origin	T0 (6 Months)	T1 (12 Months)	T2 (18 Months)	Notes on Evolution
US	Derived from pig dermis	Ten patients (16%)	Seven patients (11%)	Seven patients (11%)	Five cases of peri-capsular thickening and two cases with pseudonodular areas at T2
DM	Derived from pig dermis	Two patients (3%)	One patient (2%)	Zero patients (0%)	Radiological signs visible on DM completely disappear at T2

## Data Availability

The data presented in this study are available on request from the corresponding author. The data are not publicly available due to privacy concerns, in accordance with GDPR.
